# *Streptococcus agalactiae* Serotype Distribution and Antimicrobial Susceptibility in Pregnant Women in Gabon, Central Africa

**DOI:** 10.1038/srep17281

**Published:** 2015-11-25

**Authors:** Sabine Belard, Nicole Toepfner, Mesküre Capan-Melser, Ghyslain Mombo-Ngoma, Rella Zoleko-Manego, Mirjam Groger, Pierre-Blaise Matsiegui, Selidji T. Agnandji, Ayôla A. Adegnika, Raquel González, Peter G. Kremsner, Clara Menendez, Michael Ramharter, Reinhard Berner

**Affiliations:** 1 Centre de Recherches Médicales de Lambaréné, Hôpital Albert Schweitzer, Lambaréné, Gabon; 2 Institut für Tropenmedizin, Universität Tübingen, Tübingen, Germany; 3 Department of Pediatric Pneumology and Immunology, Charité-Universitätsmedizin Berlin, Berlin, Germany; 4 Clinic and Polyclinic for Pediatrics and Adolescent Medicine, University Hospital Carl Gustav Carus, Technische Universität Dresden, Dresden, Germany; 5 Department of Medicine I, Division of Infectious Diseases and Tropical Medicine, Medical University of Vienna, Vienna, Austria; 6 Département de Parasitologie, Université des Sciences de la Santé, Libreville, Gabon; 7 Ngounie Medical Research Centre, Fougamou, Gabon; 8 ISGlobal, Barcelona Ctr. Int. Health Res (CRESIB), Hospital Clínic- Universitat de Barcelona), Barcelona, Spain

## Abstract

Neonatal invasive disease due to *Streptococcus agalactiae* is life threatening and preventive strategies suitable for resource limited settings are urgently needed. Protective coverage of vaccine candidates based on capsular epitopes will relate to local epidemiology of *S. agalactiae* serotypes and successful management of critical infections depends on timely therapy with effective antibiotics. This is the first report on serotype distribution and antimicrobial susceptibility of *S. agalactiae* in pregnant women from a Central African region. Serotypes V, III, and Ib accounted for 88/109 (81%) serotypes and all isolates were susceptible to penicillin and clindamycin while 13% showed intermediate susceptibility to erythromycin.

Neonatal invasive disease due to *Streptococcus agalactiae* is a life threatening infection. In industrialized countries prenatal screening in pregnant women and intra-partum antibiotic prophylaxis (IAP) have been widely established and successfully reduced the incidence of newborn morbidity and mortality related to *S. agalactiae*^1^,^2^. In low income settings recto-vaginal colonization rates with *S. agalactiae* in pregnant women are similar^3^; however, screening and IAP for prevention of invasive neonatal disease is mostly not implemented due to limitations in resources and infrastructure. Vaccination of pregnant women is an alternative prevention strategy that proved effective for other neonatal infectious diseases (i.e. neonatal tetanus), and several *S. agalactiae* vaccine candidates are currently in pre-clinical and clinical development^4^. Ten capsular polysaccharides have been identified as important virulence factors of *S. agalactiae* and differentiate *S. agalactiae* into ten distinct serotypes. Based on serotype pathogenicity and prevalence, a trivalent polysaccharide-protein conjugate vaccine composed of capsular epitopes from serotypes Ia, Ib and III is in Phase II evaluation (NCT02046148); protective coverage will depend on selected *S. agalactiae* serotypes and *S. agalactiae* serotype distribution in target populations. In case of critical infections due to *S. agalactiae* effective management will depend on timely therapy with antibiotics to which *S. agalactiae* is sensitive. There is a lack of knowledge on the epidemiology of *S. agalactiae* in pregnant women from Central sub-Saharan Africa. The aim of this study was to describe serotype distribution and antimicrobial susceptibility of *S. agalactiae* in pregnant women from Gabon.

## Methods

Serotypes and antibiotic susceptibility of *S. agalactiae* isolates from HIV-negative pregnant women participating in a multicentre randomized controlled clinical trial comparing the safety and efficacy of mefloquine and sulfadoxine-pyrimethamine for intermittent preventive treatment for malaria in pregnancy (IPTp) in Gabon (MIPPAD trial; NCT 00811421, registration date December 18, 2008) were studied. Detailed information on study design, methods, clinical data and outcomes has been published recently^5^,^6^; the study protocol was approved by the CERMEL institutional ethics review board and all experiments were carried out in accordance with international regulations including the Declaration of Helsinki . In summary, recto-vaginal swabs were taken at presentation for delivery; nylon flocked swabs were used and submerged into Amies transport medium; Lim Broth was inoculated, incubated at 37 °C and then sub-cultured on Granada agar under aerobic conditions at 37 °C for 24 h; yellow-orange pigmented colonies on Granada agar confirmed growth of *S. agalactiae*. After storage at −20 °Celsius isolates were re-cultured on 5% sheep blood agar and serotypes of the capsular polysaccharides Ia, Ib, II-IX were determined by latex agglutination (Strep-B-latex kit; Article 54991/Lot La-1-4, Statens Serum Institute, Denmark) according to the manufacturer’s instructions. Isolates were additionally confirmed by *S. agalactiae* multiplex PCR as described by Creti *et al.*^7^. Antibiotic drug susceptibility testing was performed on horse blood agar plates (MHF, BioMérieux®). Disc diffusion test was performed with erythromycin (15 μg), clindamycin (2 μg), cefotaxim (30 μg), vancomycin (5 μg), and linezolid (10 μg). gMIC determination was performed as E-Test (BioMérieux®) for benzylpenicillin, cefalotin, cefotaxim and gentamicin; isolates were screened for the presence of high-level gentamicin resistance (HLGR, MIC > 500 mg/L). Isolates with intermediate susceptibility to erythromycin were investigated for inducible clindamycin resistance by double disc diffusion test (DDT). Disc diffusion tests and gMIC determination were performed and interpreted according to the EUCAST (www.eucast.org) guidelines.

## Results and Discussion

The prevalence of maternal *S. agalactiae* colonization at delivery has been reported by Capan-Melser *et al.* and was 19% (106/549; 95% CI 16–23%); *S. agalactiae* colonization was unrelated to IPTp and illiteracy was the only factor associated with higher colonization rates^5^. One hundred and nine *S. agalactiae* isolates were available for serotyping and antibiotic resistance testing. Serotyping by latex agglutination test and PCR was congruent and there was no non-typeable isolate. Almost a third (33/109, 30.3%) of isolates were serotype V; second most prevalent was serotype III (30/109, 27.5%), followed by serotype Ib (25/109, 22.9%), serotype Ia (14/109, 12.8%), and serotype II (7/109, 6.4%) (Table 1). No serotypes IV, VI, VII, XIII or XI were identified.

Serotype distribution varies between countries. Serotype V as most common serotype has been reported from The Gambia^8^ and Egypt^9^ as well, while in South Africa and Morocco serotypes Ia and III dominated with around 30–40% and serotype V represented only around 10% of isolates^10^,^11^,^12^. Two studies from Zimbabwe found serotypes III, V and Ia to be the most prevalent serotypes in decreasing order^13^,^14^. Importantly, one study from South Africa did not find significant differences in serotype distribution between vaginal and newborn colonizing isolates, but serotype distribution of invasive *S. agalactiae* isolates were significantly different to that of colonizing isolates^11^. Therefore, in the light of development of serotype-based vaccines, studies on invasive isolates are crucial. In a systematic review and meta-analysis on *S. agalactiae* disease in infants aged <3 months no low-income country was represented in the serotype assessment due to lack of data; the most frequently identified serotype in all regions with available data was serotype III (48.9%) followed by serotypes Ia (22.9%), Ib (7.0%), II (6.2%), and V (9.1%), and five serotypes (Ia, Ib, II, III, V) accounted for >85% of all serotypes^15^. Limited data on molecular characteristics of African GBS isolates suggests that the overall population structure is similar to industrialized countries^16^,^17^,^18^.

In this study, by disc diffusion test evaluation, all isolates were susceptible to clindamycin, cefotaxim, linezolid and vancomycin. Fourteen (13%) isolates showed intermediate susceptibility to erythromycin (Table 2); for these isolates no inducible clindamycin resistance was found by DDT. By E-test, no resistance to benzylpenicilline, cefotaxim, and cefalotin, and no high level gentamicin resistance were observed (Table 2). Whereas various studies in Asia, Europe and the United States have found a significant association between serotype V and macrolide resistance, it is remarkable that this association was not found in our cohort (Fisher’s Exact Test).

*S. agalactiae* has been regarded universally susceptible to penicillins and these are the antibiotics of first choice for the treatment of *S. agalactiae* infections. Data of this study confirm susceptibility of *S. agalactiae* to penicillins also for this setting; however, evidence of *S. agalactiae* with reduced penicillin susceptibility and additional resistance to other antibiotic drug classes is increasing^19^,^20^,^21^ and also reported from the African continent^22^,^23^ underlining the importance of continuous surveillance to guide empiric treatment. Erythromycin is commonly used in case of allergy to penicillins but varying rates of erythromycin resistance are reported by most studies evaluating its susceptibility. In this study intermediate susceptibility for erythromycin was seen in 13%, a rate that has also been reported for other sub-Saharan African settings^22^,^23^. As all isolates were susceptible to clindamycin in this study; clindamycin may therefore be a more suitable second-line antibiotic for *S. agalactiae* infections than erythromycin in this setting. The absence of high level gentamicin resistance supports the recommendation that neonates with suspicion of sepsis or meningitis should be treated with the combination of ampicillin and gentamicin.

In conclusion, *S. agalactiae* isolates colonizing pregnant women in Gabon showed a serotype distribution in line with other reports from Africa and a susceptibility profile suggesting penicillins as first-line and clindamycin as alternative antibiotic for infections due to *S. agalactiae*.

## Additional Information

**How to cite this article**: Belard, S. *et al.* *Streptococcus agalactiae* Serotype Distribution and Antimicrobial Susceptibility in Pregnant Women in Gabon, Central Africa. *Sci. Rep.* **5**, 17281; doi: 10.1038/srep17281 (2015).

## Figures and Tables

**Table 1:** Serotype distribution of *S. agalactiae* isolates.

Serotype	Distribution Total n = 109 [n (%)]
Ia	14 (12.8)
Ib	25 (22.9)
II	7 (6.4)
III	30 (27.5)
V	33 (30.3)

**Table 2:** Antimicrobial susceptibility of *S. agalactiae* isolates (Total n = 109 isolates).

Antibiotic Agent	Interpretation	Sensitiven (%)	Intermediaten (%)	Resistantn (%)	MIC Range(MIC50)
*E-Test*	*(MIC (mg/L))*				
Benzylpenicillin	S > 0.25, R < 0.25	109 (100)	0	0	0,016;0,19 (0.064)
Cefalotin	S > 0.25, R < 0.25	109 (100)	0	0	0.032;019 (0.125)
Cefotaxim	S > 0.25, R < 0.25	109 (100)	0	0	<0.016;0.064 (0.047)
Gentamicin	HLGR > 500mg/l	No HLGR			2;256 (48)
*Disc diffusion test*	*(Zone diameter (mm))*				
Erythromycin (15μg)	S > 21, I 18–20, R ≤ 17	95 (87.2)	14 (12.8)	0	–
Vancomycin (5μg)	S > 13, R < 13	109 (100)	NA	0	–
Linezolid (10μg)	S > 19, I 16–18, R < 15	109 (100)	0	0	–
Clindamycin (2μg)	S > 17, R < 17	109 (100)	NA	0	–

MIC: minimum inhibitory concentration, HLGR: high level gentamicin resistance.

## References

[b1] CDC. Prevention of Perinatal Group B Streptococcal Disease, Revised Guidelines (2010). Available at: http://www.cdc.gov/mmwr/pdf/rr/rr5910.pdf. (Accessed: 04.02.2015)

[b2] Lopez Sastre J. B., Fernandez Colomer B., Coto Cotallo G. D. & Ramos Aparicio A. Trends in the epidemiology of neonatal sepsis of vertical transmission in the era of group B streptococcal prevention. Acta Paediatr 94 (4), 451 (2005). 16092460 10.1111/j.1651-2227.2005.tb01917.x

[b3] Capan M. *et al.* Epidemiology and management of group B streptococcal colonization during pregnancy in Africa. Wien Klin Wochenschr 124 Suppl 3, 14 (2012). 23064861 10.1007/s00508-012-0239-5

[b4] Chen V. L., Avci F. Y. & Kasper D. L. A maternal vaccine against group B streptococcus: past, present, and future. Vaccine 31 Suppl 4, D13 (2013). 23973342 10.1016/j.vaccine.2012.12.080 PMC3757342

[b5] Capan-Melser M. *et al.* Evaluation of intermittent preventive treatment of malaria against group B streptococcus colonization in pregnant women: a nested analysis of a randomized controlled clinical trial of sulfadoxine/pyrimethamine versus mefloquine. *J Antimicrob Chemother* pii: dkv041. [Epub ahead of print] (2015). 10.1093/jac/dkv041 25722300

[b6] Gonzalez R. *et al.* Intermittent preventive treatment of malaria in pregnancy with mefloquine in HIV-negative women: a multicentre randomized controlled trial. PLoS Med 11 (9), e1001733 (2014). 25247709 10.1371/journal.pmed.1001733 PMC4172436

[b7] Creti R., Fabretti F., Orefici G. & von Hunolstein C. Multiplex PCR assay for direct identification of group B streptococcal alpha-protein-like protein genes. J Clin Microbiol 42 (3), 1326 (2004). 15004110 10.1128/JCM.42.3.1326-1329.2004 PMC356896

[b8] Suara R. O. *et al.* Carriage of group B streptococci in pregnant Gambian mothers and their infants. J Infect Dis 170 (5), 1316 (1994). 7963736 10.1093/infdis/170.5.1316

[b9] Shabayek S., Abdalla S. & Abouzeid A. M. Serotype and surface protein gene distribution of colonizing group B streptococcus in women in Egypt. Epidemiol Infect 142 (1), 208 (2014). 23561305 10.1017/S0950268813000848 PMC9152610

[b10] Kwatra G. *et al.* Serotype-specific acquisition and loss of group B streptococcus recto-vaginal colonization in late pregnancy. PLoS One 9 (6), e98778 (2014). 24979575 10.1371/journal.pone.0098778 PMC4076185

[b11] Madzivhandila M. *et al.* Serotype distribution and invasive potential of group B streptococcus isolates causing disease in infants and colonizing maternal-newborn dyads. PLoS One 6 (3), e17861 (2011). 21445302 10.1371/journal.pone.0017861 PMC3061872

[b12] Aitmhand R., Moustaoui N., Belabbes H., Elmdaghri N. & Benbachir M. Serotypes and antimicrobial susceptibility of group B streptococcus isolated from neonates in Casablanca. Scand J Infect Dis 32 (3), 339 (2000). 10879617 10.1080/00365540050166108

[b13] Moyo S. R., Mudzori J., Tswana S. A. & Maeland J. A. Prevalence, capsular type distribution, anthropometric and obstetric factors of group B streptococcus (*Streptococcus agalactiae*) colonization in pregnancy. Cent Afr J Med 46 (5), 115 (2000). 11210331 10.4314/cajm.v46i5.8533

[b14] Mavenyengwa R. T., Maeland J. A. & Moyo S. R. Serotype markers in a *Streptococcus agalactiae* strain collection from Zimbabwe. *Indian* J Med Microbiol 28 (4), 313 (2010). 10.4103/0255-0857.71819 20966561

[b15] Edmond K. M. *et al.* Group B streptococcal disease in infants aged younger than 3 months: systematic review and meta-analysis. Lancet 379 (9815), 547 (2012). 22226047 10.1016/S0140-6736(11)61651-6

[b16] Brochet M., Couve E., Bercion R., Sire J. M. & Glaser P. Population structure of human isolates of *Streptococcus agalactiae* from Dakar and Bangui. J Clin Microbiol 47 (3), 800 (2009). 19109468 10.1128/JCM.01103-08 PMC2650903

[b17] Florindo C. *et al.* Molecular epidemiology of group B streptococcal meningitis in children beyond the neonatal period from Angola. J Med Microbiol 60 (Pt 9), 1276 (2011). 21474607 10.1099/jmm.0.031674-0

[b18] Huber C. A., McOdimba F., Pflueger V., Daubenberger C. A. & Revathi G. Characterization of invasive and colonizing isolates of *Streptococcus agalactiae* in East African adults. J Clin Microbiol 49 (10), 3652 (2011). 21865428 10.1128/JCM.01288-11 PMC3187314

[b19] Andrews J. I. *et al.* Group B streptococci causing neonatal bloodstream infection: antimicrobial susceptibility and serotyping results from SENTRY centers in the Western Hemisphere. Am J Obstet Gynecol 183 (4), 859 (2000). 11035326 10.1067/mob.2000.108839

[b20] Kimura K. *et al.* High frequency of fluoroquinolone- and macrolide-resistant streptococci among clinically isolated group B streptococci with reduced penicillin susceptibility. J Antimicrob Chemother 68 (3), 539 (2013). 23111853 10.1093/jac/dks423

[b21] Crespo-Ortiz Mdel P., Castaneda-Ramirez C. R., Recalde-Bolanos M. & Velez-Londono J. D. Emerging trends in invasive and noninvasive isolates of *Streptococcus agalactiae* in a Latin American hospital: a 17-year study. BMC Infect Dis 14, 428 (2014). 25086463 10.1186/1471-2334-14-428 PMC4131052

[b22] Moyo S. R., Maeland J. A. & Munemo E. S. Susceptibility of Zimbabwean *Streptococcus agalactiae* (group B streptococcus; GBS) isolates to four different antibiotics. Cent Afr J Med 47 (9–10), 226 (2001). 12808772

[b23] Joachim A., Matee M. I., Massawe F. A. & Lyamuya E. F. Maternal and neonatal colonisation of group B streptococcus at Muhimbili National Hospital in Dar es Salaam, Tanzania: prevalence, risk factors and antimicrobial resistance. BMC Public Health 9, 437 (2009). 19948075 10.1186/1471-2458-9-437 PMC2791767

